# Determination of Cell Death Induced by Lovastatin on Human Colon Cell Line HT29 Using the Comet Assay

**DOI:** 10.17795/jjnpp-10951

**Published:** 2013-11-02

**Authors:** Marzieh Jafari, Mohsen Rezaei, Heibatullah Kalantari, Mahmoud Hashemitabar

**Affiliations:** 1 Department of Pharmacology and Toxicology, School of Pharmacy, Ahvaz Jundishpur University of Medical Sciences, Ahvaz, IR Iran; 2 Cellular and Molecular Research Center, Ahvaz Jundishpur University of Medical Sciences, Ahvaz, IR Iran

**Keywords:** Apoptosis, DNA Fragmentation, Comet Assay, HT29 Cells

## Abstract

**Background:**

Apoptosis or programmed cell death is an essential process for elimination of damaged cells. Also, induction of apoptosis is fundamental for treating cancer. Screening for agents that induce apoptosis in tumor cells help in the development of novel agents for cancer treatment. Numerous studies suggest that the exposure of tumor cells to statins can lead to cell death via two separate processes: apoptosis or necrosis. Severe fragmentation of DNA during apoptosis can be readily measured by the neutral comet assay. Migration of DNA fragments of apoptotic cells by the electrical field can produce comet-like images.

**Objectives:**

The aim of this study was to determine the type of cell death induced by lovastatin on human colon tumor cells by using the neutral comet assay and to evaluate the utility of this method for detection of apoptosis.

**Materials and Methods:**

HT29 cells were grown in DMEM medium then exposed to different concentrations of lovastatin, and DNA fragmentation associated with apoptosis was detected by the neutral comet assay method.

**Results:**

Lovastatin induced an apoptotic response in the HT29 cell line and produced a comet pattern similar to the positive control.

**Conclusions:**

This study showed that lovastatin can induce apoptosis in the HT29 cell line and confirmed the utility of comet assay for detection of apoptosis.

## 1. Background

Cell death may be described by either of the two well characterized mechanisms, apoptosis or necrosis (1). Necrosis is pathological cell death and occurs with severe damage to cells and physicochemical stress, including hypothermia, hypoxia, osmotic shock, mechanical stress and toxins. It is characterized by cell swelling, membrane degradation and release of cell contents and can lead to inflammation of the surrounding tissues. Apoptosis or programmed cell death is a physiological process that is a defined process which plays an important role in development and morphogenesis, homeostasis and elimination of damaged and harmful cells. It results in activation of apoptotic pathways, molecular and cell morphology changes such as DNA fragmentation and formation of apoptotic bodies. Apoptosis can lead to cell death, with no inflammatory effect on the neighboring cells. A wide variety of physiological and pathological stimuli can initiate apoptosis. The signal that activates downstream components of the apoptotic pathway may result from the binding of cell-surface death receptors or from damage to the genome. Dysfunction or dysregulation of the apoptotic program has implications for a variety of pathological conditions (2, 3). Alterations in control of cell death or survival are associated with pathogenesis of a variety of human diseases including cancer and many other chronic diseases.

During previous years it has been demonstrated that tumor formation can result from defects in apoptotic pathways. Most of tumor cells are resistant to apoptosis, so induction of apoptosis is fundamental for treating cancer. Many cancer therapeutic agents exert their effects through initiation of apoptosis, and even the process of carcinogenesis itself sometimes depends on the failure of apoptosis. Screening for agents that induce apoptosis in tumor cells help in the development of novel agents for cancer treatment (4, 5). Statins are lowering- cholesterol drugs and HMG-CoA reductase inhibitors (Hydroxymethylglutaryl-CoA Reductase Inhibitors). They are used for the prevention and treatment of atherosclerotic disease. The main outcome of this disease is increased levels of cholesterol. Statins act by inhibition of HMG-CoA reductase (the first and rate-limiting step in cholesterol synthesis) and blocking conversion of HMG-CoA to mevalonate and consequently inhibition of hepatic cholesterol production.

Statins show different effects, independent of their influence on cholesterol synthesis, mevalonate (metabolite of HMG-CoA reductase), is a precursor of geranyl pyrophosphate, farnesol pyrophosphate (FPP) and all-transgeranylgeranyl pyrophosphate (GGPP). These compounds bind to G proteins in the cell membrane and are involved in cell signaling and cell biological functions such as proliferation, differentiation and apoptosis. Numerous studies suggest that the exposure of tumor cells to statins can lead to cell death. Determination of cell death induced by statins can lead to new clinical application for these drugs (6-9). Several techniques have been described that detect apoptosis based on morphological and biochemical characteristics, for example propidium iodide (PI) staining, Annexin V staining and TUNEL assay. Techniques that are currently used for apoptosis detection have disadvantages and limitations and detection of apoptosis is associated with false results. Due to the importance of the apoptotic process in biological systems, the use of a diagnostic technique with high sensitivity and accuracy is important. Degradation of nuclear DNA into nucleosomal units is one of the best characterized biochemical features of apoptotic cell death (10, 11). Severe fragmentation of DNA during apoptosis can be measured by the neutral comet assay. The Comet Assay or single cell gel electrophoresis assay is one of the very widely used assays to microscopically detect DNA damage at the level of a single cell. In this assay, the shape, size and amount of DNA within the ‘comet’ play important roles in the determination of the level of damage. The massive migration of small DNA fragments of apoptotic cells by the electrical field can produce comet-like images with a long tail and a small head; while DNA fragments associated with necrosis, due to their high molecular weight, do not migrate and form a halo around the cells (12-16). This technique is highly sensitive and data collection is possible only with the use of a single cell. It also allows the use of different types of cells. Comet assay can be performed with low drug test and a large number of samples in a relatively short time can be evaluated and the results are presented both qualitatively and quantitatively.

## 2. Objectives

The aim of this study was to determine the type of cell death induced by lovastatin on human colon tumor cells by using the neutral comet assay and to evaluate the utility of this method for detection of apoptosis.

## 3. Materials and Methods

### 3.1. Cell Culture

Colon tumor cell HT29 (obtained from Pasteur Institute of Iran) were grown in DMEM medium supplemented with 10% FBS at 37˚C in humidified 5% CO_2_, 95% air incubator. For experiments, the cells were removed by trypsinization, and washed with PBS.

### 3.2. Cell Viability

Cell viability was determined before the experiments using the trypan blue exclusion test. Viable cells (cells excluding trypan blue) and nonviable cells (stained cells) were counted using a hemocytometer and cell viability was calculated.

### 3.3. Drug Treatment

The inactive lactone form of Lovastatin was converted to its active form by dissolving in ethanol and NaOH at 50˚C for 2 hours. HT29 cells (cells/mL) were exposed to different concentration of lovastatin (10, 20, 40,100 µM) for 48 and 72 hours in 6-well plates. As positive controls, cells were treated with anisomysine (2 µg/mL) for 2 hours.

### 3.4. Neutral Comet Assay

DNA fragmentation associated with apoptosis was detected by the neutral comet assay method. Slide prepration: HT29 cells, at a concentration of 10^5^ cells/mL, were mixed with 1% low temperature melting agarose (LMPA) in PBS (Phosphate buffered saline) at a ratio of 1:10 (v/v). 100 mL of this suspension was spread on a pre-coated slide and covered with a coverslip. After gelling for 10 minutes at 0˚C, the coverslip was gently removed. Lysis: slides were placed in precooled lysis solution (2.5 M NaCl, 100 mM EDTA, pH 10, 10 mM Tris base, 10% DMSO and 1% Triton X-100) at 4˚C for 30 minutes. Electrophoresis: after lysis, slides were equilibrated in TBE solution, electrophoresis was performed for 20 minutes at 25 v and 300 mA. Staining: slides were stained with ethidiumbromide solution (20 mg/mL) for 5 minutes and covered with a coverslip. Microscopic analysis: for evaluation the comet patterns, 50 nuclei from each slide were counted and comets were scored between 0-3 depending on the head size and tail length (17). The apoptotic cells were categorized by scoring them as 2 or 3. Comet of score 0 and 1 reflected intact cells.

### 3.5. Statistical Analysis

Statistical analysis was performed using the non-parametric X2 test to compare groups of data.

## 4. Results

**Figure 1.**
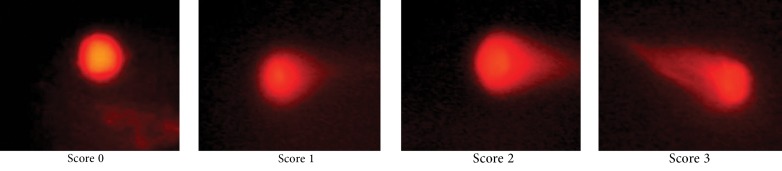
Comet Pattern of Intact (score 0, 1) and Apoptotic (score 2, 3) Cells After Exposure to Anisomycin (50X)

**Figure 2.**
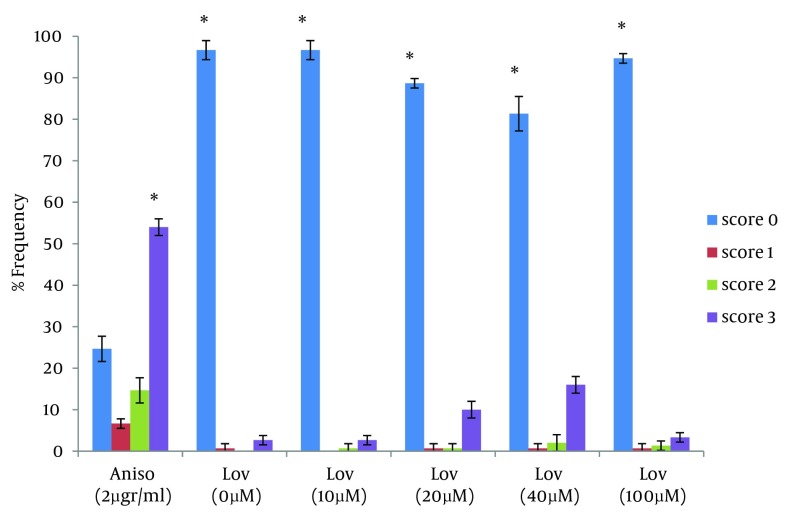
Frequency of Comet Patterns of HT29 Cells After 48 Hours of Exposure to Anisomycin and Lovastatin * P < 0.0001 statistically significant compared with the other comet patterns

**Figure 3.**
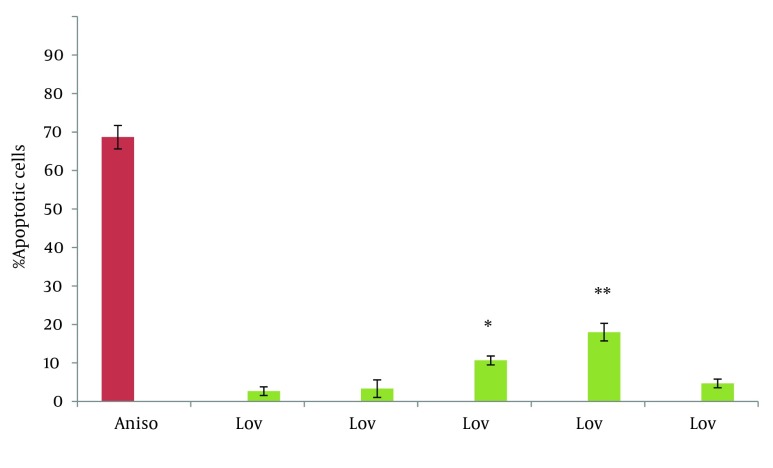
Percentage of Apoptotic Cells After 48 Hours of Exposure to Anisomycin and Lovastatin * P < 0.005, **P < 0.0001 statistically significant compared with 0, 10, 100 µM lovastatin

In this study the neutral comet assay was used for the assessment of the apoptotic effect, and exposure of cells to Anisomycin was considered as the positive control. 68.6% of cells were apoptotic after treatment with 2 µg/mL Anisomycin (Table 1). From images of the fluorescence microscope, apoptotic cells showed comet like images with long tail and small head, while intact cells were without any tail (Figure 1). Lovastatin induced an apoptotic response in HT29 cells and produced a comet pattern similar to the positive control (Anisomycin). Percentage of normal and apoptotic cells are shown in Table 1. In this study, HT29 cells were initially exposed to 10, 20, 40 µM of lovastatin for 48 hours. As shown in Table 1, Figure 2 and 3, the highest percentage of apoptotic cells was achieved at 40 µM. Lovastatin in this concentration caused an 18% apoptotic response and apoptotic cells were significantly higher than that of the negative control (P < 0.0001). Also, there were significant differences between the 20 µM sample and the negative control (P < 0.005). In the 20 µM sample, apoptotic effect was lower and 10.6 percent of cells were apoptotic. The lowest concentration of lovastatin, 10 µM had no effect at inducing apoptosis.

**Figure 4.**
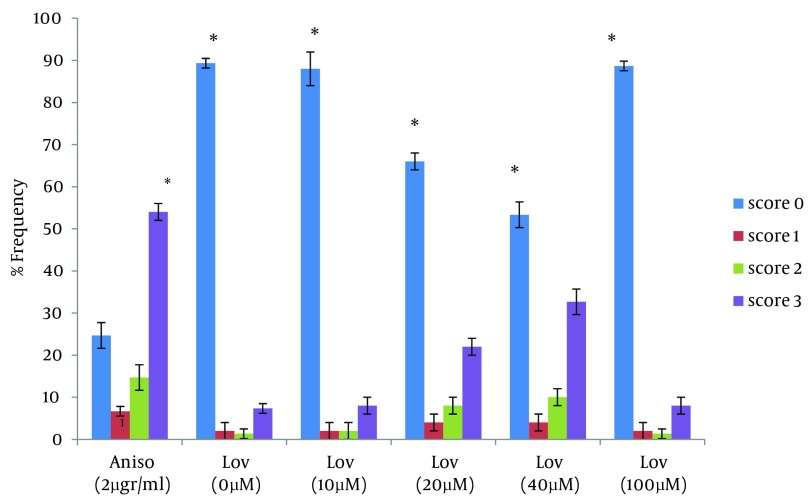
Frequency of Comet Patterns of HT29 Cells After 72 Hours of Exposure to Anisomycin and Lovastatin * P < 0.0001 statistically significant compared with the other comet patterns

**Figure 5.**
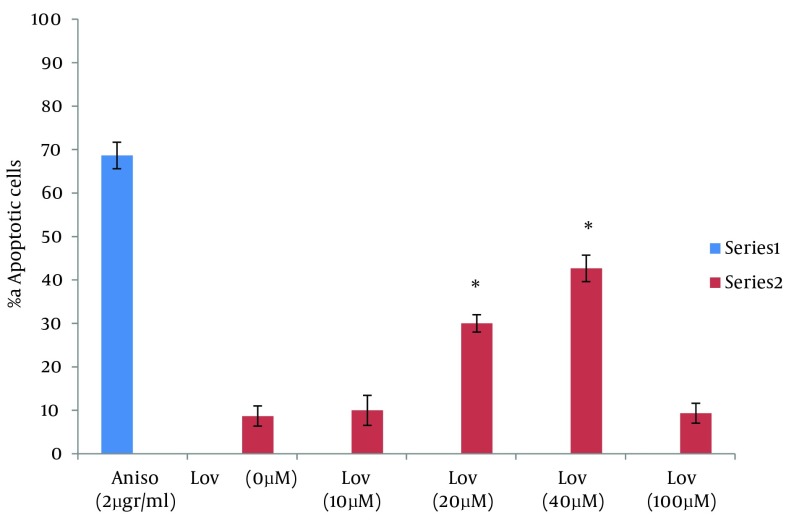
Percentage of Apoptotic cells After 72 Hours of Exposure to Anisomycin and Lovastatin * P < 0.0001 statistically significant compared with 0, 10, 100 µM lovastatin

For a more complete evaluation, HT29 cells were treated with lovastatin for 72 hours. During this time, the maximum apoptosis was observed in presence to 40 µM. This concentration caused a significant increase in apoptotic cells and percentage of apoptotic cells increased to 42.6% at 72 hours after treatment (P < 0.0001). In 20 µM, 30 % of the cells showed an apoptotic response. Similar to the 48 hours treatment, there were no significant differences between 10 µM and the negative control. (Table 1, Figure 4 and 5). We next assessed the ability of lovastatin at the concentration of 100 µM to induce apoptosis. No differences were apparent between frequency of comet patterns in this concentration and the negative control. Results were similar to the negative control group by 48 and 72 hours (Table 1, Figure 4, and 5).

**Table 1.** Percentage of Intact and Apoptotic cells After Exposure to Anisomycin and Lovastatin

	Intact Cells	Apoptotic Cells
**Anisomycin**	31.33 ± 3.04	68.67 ± 3.04
**Lovastatin, 0 µM**		
48 hours	97.33± 1.14	2.67±1.14
72 hours	91.33 ± 2.3	8.67 ± 2.3
**Lovastatin, 10 µM**		
48 hours	96.67 ± 2.3	3.33 ± 2.3
72 hours	90 ± 4.16	10 ± 3.46
**Lovastatin, 20 µM**		
48 hours	89.33 ± 1.14	10.67 ± 1.14
72 hours	70 ± 2	30 ± 2
**Lovastatin, 40 µM**		
48 hours	82 ± 4	18 ± 2.3
72 hours	57.33 ± 3.04	42.67 ± 3.04
**Lovastatin, 100 µM**		
48 hours	95.33 ± 1.14	4.67 ± 1.14
72 hours	90.67 ± 1.14	9.33 ± 2.3

## 5. Discussion

**Figure 6.**
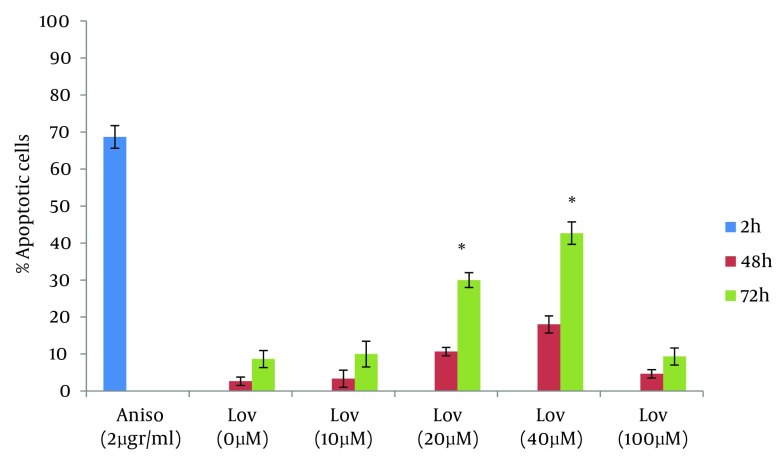
Percentage of Apoptotic Cells After 48 and 72 Hours of Exposure to Anisomycin and Lovastatin * P < 0.0001 statistically significant compared with 48h

One of the goals of cancer therapy is to induce apoptosis in cancer cells. Numerous studies suggest that the exposure of tumor cells to statins can lead to cell death. In this study we evaluated lovastatin cell death effect on human colon tumor cells by using the neutral comet assay. The results of this study are comparable with other researches on cell death effects of statins. Zhihong Jiang et al evaluated the ability of lovastatin in inducing apoptosis in glioblastoma cells. The results showed, lovastatin (5 µM) treatment, caused 22.9% cell death for glioblastoma cells at 24 hours (18). Wong et al assessed Multiple myeloma cells for their sensitivity to undergo apoptosis in response to statins. 0.53% of cells in the evaluation with propidium iodide staining and 51% of cells in TUNEL staining showed positive results after treatment with lovastatin for 48 hours (19). In this study, as noted in the results, 20 and 40 µM of lovastatin induced 10.6% and 18% of cell death in HT29 cells at 48 hours and this was less than that reported by other studies. So in order to obtain a more complete evaluation, HT29 cells were treated with lovastatin for 72 hours. Comparison of apoptosis at both 48 and 72 hours showed that apoptotic response is time dependent. Percentage of apoptotic cells with 20 and 40 µM of lovastatin at 72 hours was significantly higher than that at 48h and increased to 30 % and 42.6% (P < 0.0001) (Figure 6).

For evaluation of the lovastatin effect at higher doses, HT29 cell were exposed to 100 µM for 48 and 72 hours. For both time points, there were no significant differences from the negative control. In this context, the results from Agarwal et al studies showed lovastatin induced more apoptotic response at 100 µM than 10, 30 µM in colon tumor cells. Colon tumor cells after 48 hours of exposure to lovastatin and analysis by flow cytometry, showed a dose dependent-increased apoptotic response (20). The results of this study are in agreement with those obtained by Agarwal et al study at 10, 20, 40 µM of Lovastatin, but in contrast to Agarwal findings, this study has shown no apoptotic responses at 100 µM of lovastatin. On the other hand, Dimitroulakos et al evaluated the sensitivity of acute myeloid leukemia cells to lovastatin at 10, 50 and 100 µM. In patients with acute myeloid leukemia, the frequency of apoptotic cells in 10 and 50 µM concentration increased significantly compared to the control group. At concentration of 100 µM, different results were obtained. Apoptotic response in some patients was not significantly different from the negative control group. An increase in the percentage of apoptotic cells was observed in acute myeloid leukemia patients (21). In summary, the results of this study show that lovastatin can induce programmed cell death in colon tumor cells at 20 and 40 µM. Concentrations at 10 and 100 µM could not induce apoptosis in HT29 cells. . It was predictable lovastatin at 10 µM could not induce apoptosis, but for 100 µM, this result was not expected. Also the comet assay is an applied technique for detection of apoptosis.
